# Complement Activation in Inflammatory Skin Diseases

**DOI:** 10.3389/fimmu.2018.00639

**Published:** 2018-04-16

**Authors:** Jenny Giang, Marc A. J. Seelen, Martijn B. A. van Doorn, Robert Rissmann, Errol P. Prens, Jeffrey Damman

**Affiliations:** ^1^Department of Pathology, Erasmus Medical Center Rotterdam, Rotterdam, Netherlands; ^2^Department of Nephrology, University Medical Center Groningen, Groningen, Netherlands; ^3^Department of Dermatology, Erasmus Medical Center Rotterdam, Rotterdam, Netherlands; ^4^Center for Human Drug Research, Leiden, Netherlands

**Keywords:** complement, dermatology, skin diseases, innate immunity, psoriasis, hidradenitis suppurativa, lupus erythematosus, bullous pemphigoid

## Abstract

The complement system is a fundamental part of the innate immune system, playing a crucial role in host defense against various pathogens, such as bacteria, viruses, and fungi. Activation of complement results in production of several molecules mediating chemotaxis, opsonization, and mast cell degranulation, which can contribute to the elimination of pathogenic organisms and inflammation. Furthermore, the complement system also has regulating properties in inflammatory and immune responses. Complement activity in diseases is rather complex and may involve both aberrant expression of complement and genetic deficiencies of complement components or regulators. The skin represents an active immune organ with complex interactions between cellular components and various mediators. Complement involvement has been associated with several skin diseases, such as psoriasis, lupus erythematosus, cutaneous vasculitis, urticaria, and bullous dermatoses. Several triggers including auto-antibodies and micro-organisms can activate complement, while on the other hand complement deficiencies can contribute to impaired immune complex clearance, leading to disease. This review provides an overview of the role of complement in inflammatory skin diseases and discusses complement factors as potential new targets for therapeutic intervention.

## Introduction

### The Complement System

**Figure 1:**
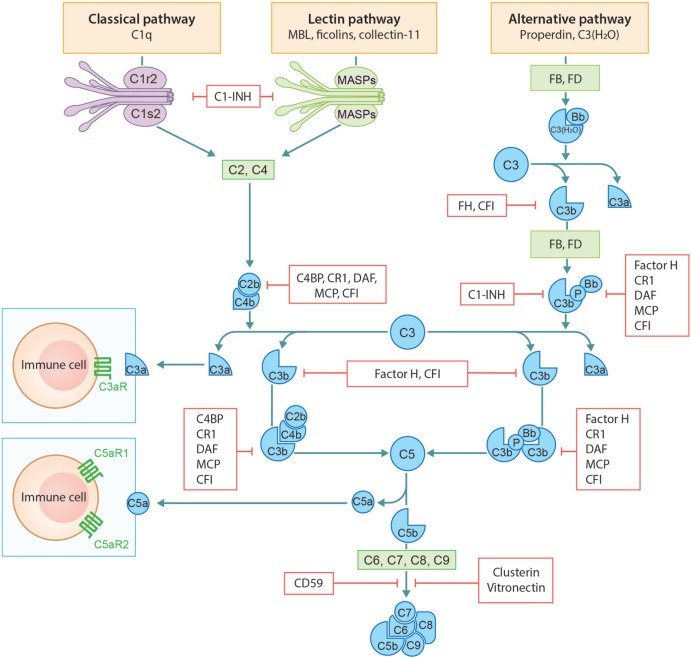
Schematic overview of the complement system: activation and regulation.

The complement system consists of a network of more than 50 different plasma and membrane-associated proteins. It is a part of the innate immune system and plays a key role in host defense against pathogens as well as in tissue homeostasis. The complement system can be activated through three distinct pathways: the classical, lectin, and alternative pathway (Figure 1). Classical pathway activation occurs after binding of the first component C1q to antibody-antigen complexes, cell particles, or certain acute phase proteins such as C-reactive protein or serum amyloid P. Once the complement activation cascade is initiated, the attached serine proteases C1r and C1s become activated. This is followed by cleavage of, respectively, C4 and C2, leading to the formation of a C3 convertase (C4b2a) that can activate C3 (1). The lectin pathway is activated when mannan binding lectin (MBL), ficolins, and/or collectins interact with carbohydrate structures predominantly present on invading pathogens. Consequently, this activates MBL-associated serine proteases 1 and 2 (MASP1 and MASP2), subsequently cleaving C4 and C2. The classical and lectin pathways generate the shared C3 convertase C4b2a, which in turn generates the opsonin C3b and anaphylatoxin C3a through cleavage of native C3. In contrast to the classical and lectin pathways, the alternative pathway is activated by low grade spontaneous hydrolysis of systemic native C3 (“C3 tickover”) or *via* properdin binding to certain cell surfaces (e.g., bacteria). Hydrolyzed C3 [C3(H_2_O)] can associate with Factor B (FB), which is subsequently activated by Factor D. This results in the formation of a C3 convertase (C3H_2_0Bb) that can finally cleave C3. This fluid phase C3 convertase cleaves C3 to generate C3a and C3b. C3b can covalently bind to nearby structures and provides the basis for generation of the surface bound C3 convertase C3bBb. Alternative pathway activation can also be initiated as an amplification loop when C3b, generated by either one of the three pathways, is deposited on the triggered surface and binds to FB, which also results in the formation of the surface bound C3 convertase C3bBb. C3 convertases can be stabilized by factor P (properdin). C3b, generated by the classical, lectin, or alternative pathway, combines with the C3 convertases to form the C5 convertases (C4b2aC3b and C3bBbC3b), initiating the terminal complement cascade. C5 convertases cleave C5 into C5b and anaphylatoxin C5a, eventually resulting in the assembly of C5b-9 by combining C5b with C6-C9. C5b-9 can form channels into the cell membrane causing cell lysis. In addition to the capacity to generate C5b-9, complement split products iC3b and C3dg are known to have immunomodulating functions (1–3).

Several soluble and cell-bound regulators are capable of mediating complement regulation on different levels of the complement cascade to limit damage to self-cells (Figure 1). C1-esterase inhibitor (C1-INH), C4b binding protein (C4BP), FH, FI, vitronectin, and clusterin are the soluble regulators. However, if complement components still manage to deposit on the cell membrane, inactivation of complement can be achieved by cell-bound regulators. These include decay-accelerating factor (DAF/CD55), membrane cofactor protein (MCP/CD46), complement receptor-1 (CR1), and CD59 (protectin) (4).

### Complement System and the Skin

Plasma complement proteins are predominantly synthesized in the liver by hepatocytes, although extrahepatic complement can also be produced by other cell types such as endothelial cells, epithelial cells, and immune cells. Furthermore, complement receptors and membrane regulators can also be synthesized by other types of cells, such as leukocytes, fibroblasts, adipocytes, and endothelial cells (5). An important role of extrahepatic synthesized complement components is the protection against micro-organisms and inflammation at tissue or organ level.

The skin is the body’s largest organ that serves as an immune and physical barrier against pathogenic organisms, irritant, xenobiotics, allergens, UV-irradiation, and mechanical injury. The epidermis is an active immune organ equipped with immune-competent cells, including Langerhans cells, keratinocytes, dendritic epidermal T-lymphocytes, and melanocytes, of which the keratinocyte is the predominant cell type. The dermis harbors many immune cells such as dermal dendritic cells (DC) and mast cells. Multiple reports have demonstrated that human keratinocytes are able to produce several complement proteins including complement components C3, C4, and FB of which synthesis can be regulated and enhanced by interleukin- 1α (IL-1α), interferon-γ (IFN-γ), and tumor necrosis factor-α (TNF-α). Furthermore, keratinocytes can also synthesize soluble complement regulators such FH and FI, complement receptors CR1, cC1qR, C5aR1, and CR2 and cell-bound complement regulator proteins MCP, DAF, and CD59. Importantly, IFN-γ can enhance the production of FH and FI locally, thereby preventing epidermal damage which could be caused by locally produced C3, C4, and FB (6, 7). Besides keratinocytes, also melanocytes express cell-bound complement regulator proteins DAF, MCP, and CD59, making these cells less vulnerable to autologous complement attack. For example in vitiligo, melanocytes and keratinocytes seem to express less cell-bound regulators, which adds to the cells susceptibility to lysis (8). Langerhans cells and DC are the antigen-presenting cells, which have the capacity to initiate primary immune responses. Human DCs are able to produce most of the complement components and are also qualified to identify soluble and cell-bound complement effector molecules. Interestingly, the functional development of DCs depends on complement production and activation. Hence, T-cell differentiation can be indirectly determined by complement, in particular the anaphylatoxins C3a and C5a (5).

The skin is colonized by a diversity of microbes including commensals and potential pathogens. The skin plays an important role in restraining the invasion of opportunistic or pathogenic organisms. The complement system may partly impact this microbial ecosystem by regulating complement C5aR1 signaling. Inhibition of C5aR1 signaling results in lower microbial diversity, making the skin less resistant to pathogenic organisms (2). Moreover, inhibiting C5aR1 signaling also decreases expression of various chemokines, cytokines, anti-microbial peptides, and pattern recognition receptors (2). In particular, downregulation of IL-12 by complement might impact the differentiation and development of T cells (9).

Overall, the complement system forms an important bridge between the innate and adaptive immune system. Complement activation products are known to mediate chemotaxis, opsonization, and mast cell degranulation, which can contribute to the elimination of pathogenic organisms. Aberrant expression and genetic deficiencies of complement components or regulators are associated with certain skin diseases. Complement activation in different skin diseases has experienced a first period of high-level attention already in the 1970s and 1980s. However, in the following decades the interest in complement has declined, paralleling intensified research into lymphocyte function, adaptive immunity, and immunogenetic associations in skin diseases. The introduction of immunosuppressive agents targeting the adaptive immune system, for example, cyclosporine in the treatment of psoriasis, shifted the interest from innate to adaptive. A second “wave of attention” in complement occurred after the development and introduction of several new complement inhibiting agents. These complement inhibitors are now effectively being used in clinical practise in several diseases, for example, in the treatment of renal disease. The main purpose of this review is to provide an overview of the role of complement in inflammatory skin diseases and to discuss the rationale and potential targets for pharmacological intervention using complement inhibiting therapeutics.

## Psoriasis

**Figure 2:**
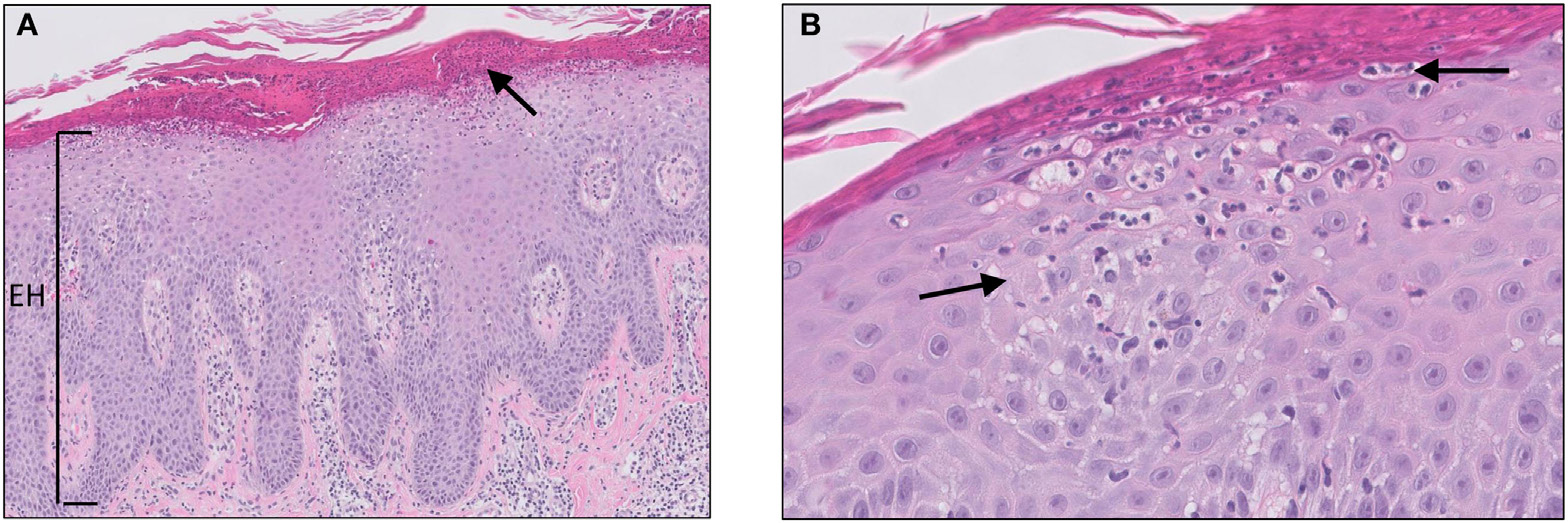
Psoriasis vulgaris. Confluent parakeratosis, psoriasiform epidermal hyperplasia [**(A)**, EH], hypogranulosis, and influx of numerous neutrophils in the corneal layer [**(A)**, arrow]. **(B)** Transepidermal migration of neutrophils from the dermis to the corneal layer (arrows).

Psoriasis is a chronic skin disease, affecting approximately 2–4% of the Western population. Although the exact etiology of psoriasis remains unclear, it is considered a multifactorial disease with genetic, environmental, and behavioral factors playing a role in the pathogenesis and course of the disease. Psoriasis vulgaris (plaque psoriasis) is the most common form of the disease (affecting 70% of the patients) and usually presents as symmetrical erythematous papules or plaques covered with thick silvery scales located on the extensor side of the elbows and knees, scalp, and lumbosacral area. The scales are a consequence of a hyperproliferative epidermis, which is reflected on histology by parakeratosis, alternating regular hyperplasia with elongation of the rete ridges, loss or absence of the granular layer, and dilated capillaries of the papillary dermis (Figure 2). In addition, the epidermis may show transepidermal migration of neutrophils, and to a lesser extent lymphocytes, to the corneal layer that results in the formation of Munro microabscesses (10, 11). A Munro microabscess is a collection of neutrophils in the stratum corneum and can be considered as one of the characteristic histological findings of psoriasis. Inflammation in the dermis is characterized by a superficial perivascular infiltrate of lymphocytes.

Already in the 1970s and 1980s, several research groups have examined the scale extracts from psoriatic lesions, unraveling the underlying mechanisms of transepidermal leukocyte migration, and Munro microabscess formation. It was found that psoriatic scales exhibit chemotactic and chemokinetic properties compared with scales from non-psoriatic patients (12, 13). Subsequently, the presence of C3a in the extracts of psoriatic scales was demonstrated (14). Deposition of C3b in the presence of immunoglobulins in stratum corneum of skin biopsies from psoriasis patients suggested activation of the classical pathway of complement (15). Furthermore, also C5a was found in psoriatic leukotactic factor (PLF) and appeared to be the most potent chemotactic factor in psoriatic scales. Several subsequent studies were able to confirm these findings by monitoring elevated levels of anaphylatoxins (C3a, C4a, C5a, C5a des-Arg) in scales of psoriatic lesions (16–19). Deposition of complement and the presence of complement activation products in psoriatic scales might be explained by locally produced and activated complement. In addition, systemic derived complement components and/or systemic complement activation could also be an important source of complement deposition in psoriasis.

Regarding systemic complement activation, sera, and plasma from psoriasis patients showed elevated levels of complement components, complement split products, and regulator proteins including FB, Bb, C3, C3a, C3b, C4, C4a, C4d, C5b-9, C1-INH, C4BP, FH, and FI. These results suggest involvement of both classical and alternative pathways in systemic complement activation (20–24). Marley et al. demonstrated low-circulating properdin levels in psoriatic patients, also indicating alternative pathway activation (25). Ohkohchi et al. found that, although circulating levels of classical complement pathway regulators C1-INH and C4BP were slightly increased, marked increase of alternative pathway regulator proteins were found and associated with disease activity and skin involvement (23). Altogether, these findings support a prominent role for systemic alternative pathway activation in psoriasis. However, the most convincing and controlled study was performed by Fleming et al., taking into account and excluding confounders that could potentially bias results from the previously described studies. These included discrepancy in the involved pathways, infection, and different types of psoriasis. It was found that circulating C5b-9 levels did not correlate with the extent and disease activity. More importantly, the absence of specific associated components, such as the immune complexes C1r-C1s-C1-INH and C3bBbP, in the plasma of psoriasis patients with elevated C5b-9 levels is a direct evidence that systemic complement is not activated in psoriasis (20).

Thus, previously cited literature shows that local complement is activated in psoriatic scales but systemic complement is unlikely to be activated in psoriasis. Therefore, it is reasonable to assume that complement activation products in the circulation originate from spillover of local products into the circulation (26). Important questions in this respect are: what are the triggers for local complement activation and what is the overall contribution of complement activation in the pathogenesis of psoriasis.

There are several hypotheses that might explain the activation of complement and the inherent generation of C5a in psoriatic scales. Firstly, natural auto-antibodies are directed against carbohydrate antigens in the stratum corneum and this results in IgG, C3b, and/or C4b deposition, reflecting classical pathway activation (27, 28). However, one study provided evidence that IgG and C3b binding to stratum corneum also occurred in other parakeratotic lesions such as verruca vulgaris and lichen simplex chronicus. Yet these parakeratotic lesions do not evolve into psoriatic lesions, suggesting that immunoglobulin binding and complement activation within the lesional stratum corneum might not be a consequential pathogenic event in psoriasis (15). Secondly, complement is activated independent of immunoglobulins *via* direct cleavage of C5 by serine proteinases present in the stratum corneum or *via* alternative pathway activation. The alternative pathway can be activated when serum comes in direct contact with the stratum corneum after traumatic injury (29, 30). Thirdly, complement is activated by microbial products, which is supported by the fact that infections can trigger the exacerbation of psoriasis in a much higher number of patients than was previously believed (26, 31). Lastly, complement is activated in the dermis, pervades into the epidermis, causing neutrophils to migrate, and damage epidermal cells (32). Although previously cited studies have shown local complement activation in psoriatic scales, one should argue what is the source of complement in the upper epidermis. In the literature, there have been several reports of keratinocytes being a rich source of producing complement components including C3, FB, FH, and FI. Regarding the terminal complement components (C5–C9), a study revealed keratinocytes to express and release C7 and C9 (33). Moreover, both TNF-α and IFN-γ have been shown to augment the production of C3 by keratinocytes (34). Although systemic complement *activation* is unlikely to play a role in psoriasis as previously described, circulating complement components could also be a rich source for local activation.

Whatever the cause of complement activation in psoriasis might be, it is interesting to speculate about the concept that complement might be one of the initiating factors in the pathogenesis of psoriasis. Psoriasis is regarded as an auto-immune disease and therefore therapy in psoriasis has mainly focused on targeting the adaptive immune system. Clinical and mechanistic studies are generally conducted in patients with stable plaque type psoriasis (psoriasis vulgaris), characterized by an adaptive T-cell predominant phenotype. However, to study the pathogenesis of psoriasis, one has to take into account the stage, activity, and type of psoriasis. Early psoriatic lesions are characterized clinically by pinpoint papules, papulopustular lesions or as pustular psoriasis. Histologically, early psoriatic lesion show influx of neutrophils in the dermal papillae, transepidermal migration of neutrophils (squirting papillae), dermal capillary activation, tortuous dilatation, and angiogenesis (35–37). Importantly, this stage is characterized by predominance of neutrophils, in particular in pustular psoriasis. The pathogenesis is auto-inflammatory and driven by innate immune activation, in contrast to lymphocyte predominant auto-immune activation in stable plaque type psoriasis. In the complex immunopathology of psoriasis, it is thought that during innate immune activation, plasmacytoid dendritic cells (pDCs) are activated together with IL-1β and TNF-α producing keratinocytes in response to dendritic Toll-like receptor activation by DNA (LL-37). In this initiating phase, neutrophil recruitment has mainly been attributed to the release of IL-1β and TNF-α. However, anaphylatoxin C5a is the most potent chemo-attractant for various inflammatory cells including neutrophils, monocytes, and macrophages. It was hypothesized that in the earliest psoriatic neutrophil predominant lesions, complement is activated in the stratum corneum, C5a is released which can activate immune cells and keratinocytes. C5a can migrate to the dermal microvasculature, activating endothelial cells, and mast cell degranulation, which results in capillary tortuous dilatation, so characteristic for (early) psoriasis. To investigate this hypothesis, Tagami et al. analyzed the effect of intradermal PLF (mainly consisting of C5a) injections in guinea pigs. Light microscopy of skin biopsies revealed features reminiscent of actual psoriasis lesions and comparable with injection of C5a *in vivo* in healthy human volunteers (38). These histological features included dense infiltration of neutrophils in the dermis followed by increased epidermopoiesis, epidermal proliferation, and degeneration. In contrast, PLF/C5a did not induce proliferation or influenced the viability of cultured human epidermal cells (32). These results suggest that PLF/C5a requires specific circumstances necessary to exert its effect on skin, most probably the presence of neutrophils from the circulation. Altogether, besides IL-1β and TNF-α, C5a is likely to play a role in the early neutrophil predominant and auto-inflammatory phase of psoriasis. Following auto-inflammatory initiation, the response is shifted toward auto-immune activation including activation of the IL-23/IL-17 axis characteristic for stable plaque-type psoriasis. However, more and more evidence points toward bimodal immune activation in psoriasis. Alternating waves of auto-inflammatory bursts of neutrophils coexist with T-cell driven auto-immune activation (37). Complement might be one of these factors linking innate auto-inflammatory and adaptive auto-immune responses in psoriasis. Two recent studies have provided evidence supporting this theory. Treatment of mice with siRNA targeting C3 reduced skin disease in a mouse model of psoriasis (39). Furthermore, using the imiquimod-induced model of psoriasis, it was found that C3 deficient mice showed significantly reduced levels of IL-1β, TNF-α, IL-17a, and IL-23 in the skin and draining lymph nodes and decreased infiltration of neutrophils (40). These studies clearly show a link between complement activation and activation of IL-23/IL-17 axis of adaptive immunity. Clinical studies targeting complement activation might investigate whether flares of psoriasis can be prevented by targeting innate immunity instead of treating adaptive immune activation in well-established psoriatic plaques.

## Acne Vulgaris and Hidradenitis Suppurativa (HS)

### Acne Vulgaris

Acne vulgaris is a common cutaneous disorder and its pathogenesis is multifactorial including genetic, infectious, and hormonal factors. The distribution of the skin lesions in acne vulgaris reflects that of sebaceous glands. Patients present with comedones predominantly on the face, nose, forehead, and chest (sebaceous areas). Light microscopy reveals open and closed comedones, which are a result of excessive sebum secretion, hyperkeratosis of the sebaceous duct, and follicular infundibulum, subsequently followed by hair follicle dilatation. Excessive dilatation of the follicular infundibulum eventually results in rupture of the epithelial layer. Besides distension, epithelial damage is also attributed to overgrowth of *Propionibacterium acnes* (*P. acnes*) in the hair follicle lumen. *P. acnes* is an anaerobic bacterium and a habitual follicular resident. Although data exist implicating *P. acnes* in the initiation of follicular distension and obstruction, the bacterium is primarily involved in the subsequent inflammatory response of the hair follicle. Once ruptured, secondary inflammatory changes occur, such as granulomatous inflammation in response to epithelial fragments, and chronic-active inflammation (41, 42).

Today, it is still not known what is the sequence of events that is responsible for evolution of a non-inflamed into an inflamed acne lesion. Evidence exists that complement activation could be one of the primary triggers in this inflammatory response in acne. In 1966, Puhvel et al. revealed that high levels of complement fixing antibodies are present in patients with severe acne compared with patients with mild or no acne (43). Scott et al. analyzed non-inflamed (white- and black heads) and inflamed (papules and nodules) acne lesions. The most frequent finding was perivascular granular and/or linear pilosebaceous basement membrane zone (of affected units) deposition of C3b in both non- and inflamed acne lesions (44). Similar findings were reported by Dahl et al. in early acne lesions (12). In non-inflamed acne lesions, C3b deposition preceded influx of mononuclear cells while inflamed lesions showed both C3b deposition as well as mononuclear inflammation. Kligman et al. observed that microscopic hair follicle rupture precedes clinical inflammation. In uninvolved skin of patients with acne sometimes microcomedones are seen with early inflammatory changes, with migration of neutrophils into intact follicular epithelium. In a later stage, the follicular epithelium becomes spongiotic and small foci of neutrophils can be found in the hair follicle lumen (45). These findings suggest that in the early stages of acne chemotactic factors are released from the intact hair follicle to recruit neutrophils. Complement activation products, in particular C5a, is the most likely factor involved in the chemokinesis of neutrophils that initiates the conversion of a non-inflamed into an inflamed lesion. Interestingly, C3b deposition was almost exclusively found without concurrent immunoglobulin or C1q deposition, suggesting that the alternative pathway might be primarily involved in the development of acne vulgaris. A candidate triggering factor for complement activation might be *P. acnes*, since bacteria are well known to activate complement. It has been shown that both the alternative and classical complement pathways can be activated in normal human serum upon interaction with comedonal contents as well as *P. acnes* (46–49). Another possibility is the direct activation of the alternative pathway by the stratum corneum (30). Both Dahl et al. and Scott et al. only found isolated granular C3b deposition or granular C3b with immunoglobulin deposition along the dermo-epidermal junction in a few biopsies. This could hypothetically represent immune complex formation with *P. acnes* and possibly subsequent classical pathway activation (12, 44). Whatever the trigger or route of complement activation might be, C5a is released acting as a potent chemokinetic factor in the recruitment of neutrophils. Therefore, it is plausible to hypothesize that the increased influx of neutrophils in acne vulgaris is, at least in part, dependent on complement activation and may therefore be amenable to complement inhibiting therapies.

### Hidradenitis Suppurativa

Hidradenitis suppurativa (synonym: acne inversa) is a severe inflammatory follicular skin disease causing severe patient discomfort and psychosocial burden. HS is a common, yet unrecognized and underdiagnosed disease with a prevalence of 1–4% of the European population. Patients present in the acute stage with painful inflamed nodules (boils) in the inverse apocrine bearing regions of the body such as the groin and axilla. In a late stage, sinus tract formation occurs with formation of abscesses and scarring. Importantly, systemic therapy with immunosuppressive agents (systemic corticosteroids, dapsone, and cyclosporin) has been investigated in the past decade and has shown limited efficacy.

**Figure 3:**
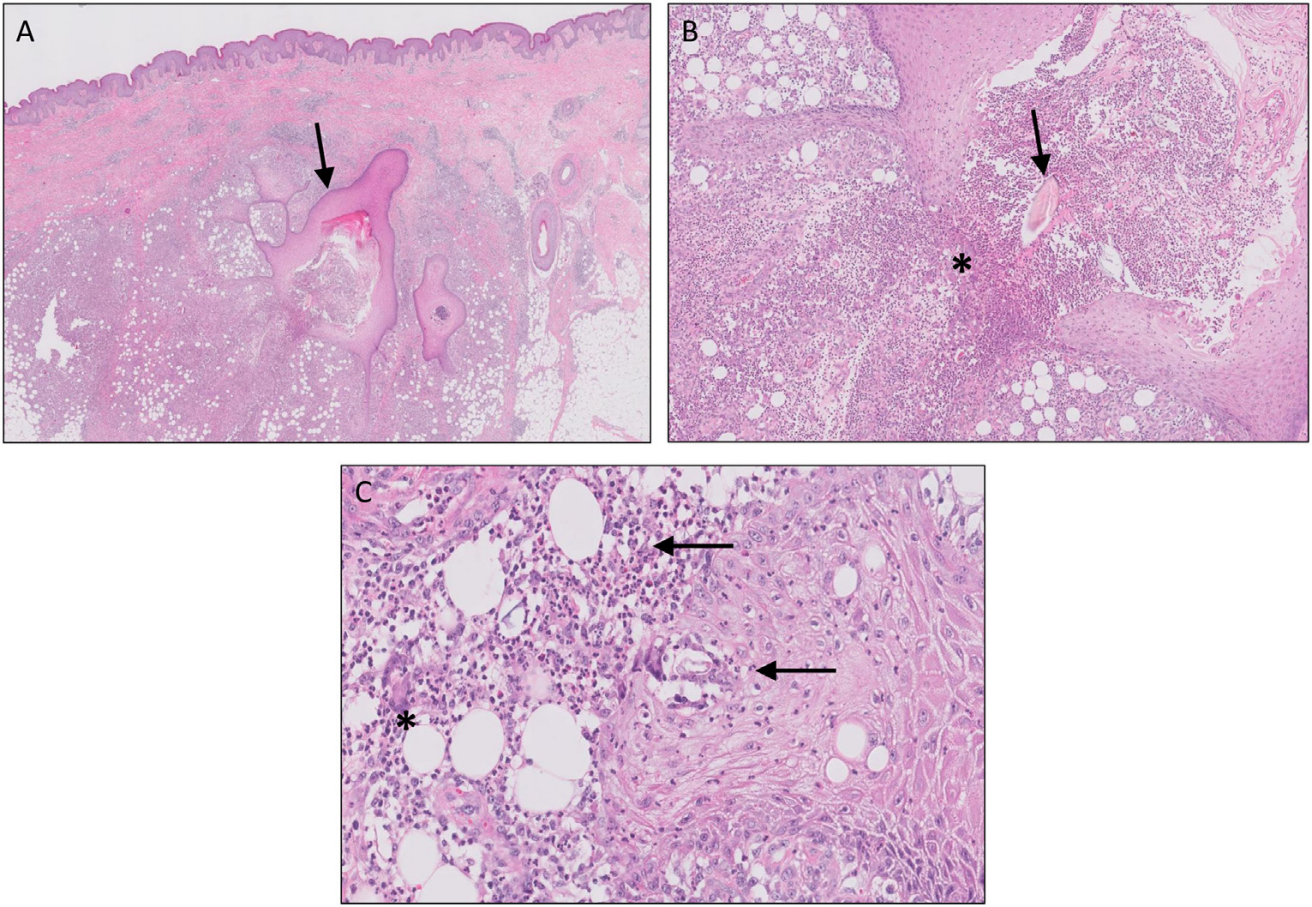
Hidradenitis suppurativa. Sinus tracts [**(A)**, arrow], active inflammation, and rupture [**(B)**, asterisks] of the follicular epithelium with “floating” hair fragments [**(B)**, arrow]. Subsequently, a secondary inflammatory response is induced with influx of numerous neutrophils [**(C)**, arrows] and granulomatous foreign body reaction with giant cells [**(C)**, asterisks].

Although the exact pathogenesis of HS still has yet to be unraveled, different theories have been proposed during the last decade. In general, it is believed that HS is a multifactorial disease in which genetic, environmental factors (such as smoking, microbial colonization, and obesity) and the immune system interact. Follicular occlusion is thought to be the primary event in HS and is caused by follicular infundibular epithelial hyperplasia and hyperkeratosis. It is hypothesized that this could be a result of microbial overgrowth due to a deficient follicular skin immune system or, in contrast, an overactive immune system that reacts to normal skin flora. Eventually, follicular plugging and occlusion will result in hair follicle rupture. Upon rupture, classical histological features are observed as seen in a ruptured epidermal cyst or comedone such as granulomatous inflammation on keratin fibers and chronic inflammation (Figure 3). Although such material is rapidly cleared in ruptured epidermal/acne cysts, the inflammatory response in HS persists, leading to chronicity with formation of sinus tracts and scarring (50–52).

Although “HS” is a misnomer since involvement of sweat glands is not a central event in the pathogenesis of HS, HS is indeed characterized by suppuration with deep abscess formation. Although not classified as a neutrophilic dermatosis, influx of numerous neutrophils is a characteristic histological feature of HS. However, it is uncertain whether this is an early or late event in the pathogenesis of this disease. The inflammatory response in HS is characterized by increased levels of IL-1β and TNF-α, both in (peri)lesional skin biopsies as well as circulating levels in HS patients (53). The important role of these cytokines is illustrated with the clinical improvement observed after treatment with TNF-α antagonists infliximab and adalimumab (54). Recruitment of neutrophils has been mainly attributed to TNF-α and IL-1β, however, the role of complement activation has only recently been studied. Blok et al. found induction of complement pathway genes in lesional HS skin biopsies, indicating involvement of the complement system in this disease (55). An open label phase 2 study is currently underway with the C5a inhibitor IFX-1 in patients with moderate to severe HS (NCT 03001622). Recent results from this study demonstrated systemic complement activation in HS patients reflected by increased circulating levels of C5a and C5b-9 (56). Furthermore, increased C5a serum levels in HS patients were able to activate neutrophils, thereby contributing to HS symptoms. Up to 83%, HS clinical response rate was achieved at the endpoint of anti-C5a treatment in this phase 2 clinical study. Thus, targeting the C5a–C5aR axis may represent a promising therapeutic strategy for HS patients, most probably *via* inhibition of systemic neutrophil activation. Besides systemic activation, also local complement could potentially be activated in HS since commensal follicular skin bacteria, similar to acne vulgaris, might function as pathogen-associated molecular patterns that can activate classical and alternative complement pathways. Furthermore and in a later stage after hair follicle rupture, cellular fragments might function as danger-associated molecular patterns, which can also activate complement. Whether complement activation, and aberrant immune activation in general in HS, is a primary or secondary event to an initial hair follicle process remains to be investigated.

## Systemic and Cutaneous Lupus Erythematosus (LE)

Lupus erythematosus is a heterogeneous auto-immune disease, which is characterized by the presence of elevated immune complexes, high titers of auto-antibodies against nuclear and cytoplasmic components and consumption of complement components. Deposition of immune complexes within various organs causes tissue damage, producing a broad spectrum of clinical manifestations ranging from systemic to solely cutaneous lesions. Epidemiological studies have estimated the prevalence of systemic LE (SLE) to be around 0.2–0.7% occurring predominantly in patients older than 40 years of age, with a female/male ratio of 9:1 (57). The cause of SLE is multifactorial with genetic, environmental, and hormonal contributions, also integrated in the American College of Rheumatology (ACR) guideline for SLE. This guideline requires at least four of the 11 clinical and laboratory components to be present before diagnosing SLE. Whereas the revised classification Systemic Lupus International Collaborating Clinics requires four components with one clinical and one immunologic item (58). Studies have shown that skin involvement will eventually develop in about 70% of patients with SLE, although primary cutaneous LE (CLE) mainly develops in the absence of systemic features. The probability of progression from CLE to SLE is approximately 20% (59). There are a few subsets in which CLE can be classified: acute LE, subacute (SCLE), and chronic cutaneous LE (CCLE), of which CCLE can be further subdivided into discoid LE, LE profundus, LE tumidus, chilblain lupus, and bullous LE (60).

Literature over the years suggests that the pathogenesis of SLE and CLE might share common features, along with the developmental consequences of genetic, environmental factors, and immune dysregulation. Several haplotypes and certain alleles of the major histocompatibility complex (MHC) have shown association with different subtypes of CLE. From a mechanistic point of view, MHC polymorphisms may lead to an increase of auto-reactive T-lymphocytes mediated by a selection error in the thymus (61). Furthermore, a panel of single nucleotide polymorphisms (SNPs) has been shown to be associated with CLE, including TYK2, IRF5, and CTLA4. These polymorphisms may have an influence on the IFN cytokine signaling (62).

A well-known trigger of CLE is ultraviolet light (UVL), also one of the ACR criteria of SLE, included as photosensitivity. Firstly, UVL can penetrate through different layers of the skin, causing acute inflammation as well as damage to the DNA, thereby inducing keratinocyte apoptosis (63). Accumulation of apoptotic material due to decreased clearance can result in the formation of immune complexes, which in turn can enhance the IFN-α production by pDCs. IFN-α is an important cytokine in the recruitment of Th1 and cytotoxic T cells (62). Secondly, UVL directly increases the production of TNF-α, interferons, and several interleukins of keratinocytes and dermal fibroblasts. These cytokines are responsible for the infiltration of leukocytes in skin biopsies of CLE (61).

Numerous auto-antibodies, resulting from impaired clearance of immune complexes, can be found in patients with CLE. The role of auto-antibodies in CLE is not completely understood, although some auto-antibodies have been found to be useful indicators in the prognosis of the disease. Patients with anti-RNP, anti-Sm, and anti-aPL antibodies are closely associated with high prevalence of malar rash, while patients with only anti-Ro/SSA antibodies demonstrated to have an increased risk for nephritis (62).

Besides genetic variation in certain MHC class I and II alleles, congenital deficiencies of classical complement pathway components C1q, C1r, C1s, C4, and C2 are strongly associated with the development of SLE (62). In addition, a homozygous SNP of the C1QA gene appears to be highly associated with SCLE (64). Boeckler et al. were the first to demonstrate a high prevalence of partial deficiency of C2, C4 and combined C2/C4 in patients with CLE (65). Furthermore, C4 copy number variation of *C4A* and *C4B genes* is also associated with the risk of SLE (extensively reviewed elsewhere) (66). Two concepts could potentially explain the development of SLE in patients deficient in classical pathway components. Firstly, early classical pathway components are involved in the induction of immune tolerance in germinal centers of lymph nodes. It is well known that CD35/CR1 and CD21/CR2 on follicular DC bind and present complement factors to virgin B-cells in order to differentiate self from non-self B-cells. More specifically, it was demonstrated that CD21, CD35, or C4 deficient mice resulted in high levels of anti-nuclear antibodies and severe lupus-like disease (67). Therefore, complement deficiency results in the breach of self-tolerance and subsequently the development of SLE. Secondly, complement deficiency results to the inability of efficiently clearing apoptotic cells/debris and could render them to become auto-antigens, induce auto-antibody formation and thereby SLE. Most research has been performed on the latter concept, in particular the role of C1q deficiency in LE. There are three sets of findings that link C1q to the development of SLE. Firstly, congenital C1q deficiency is the strongest genetic risk factor known for the development of SLE (68). Secondly and paradoxically, circulating levels of C1q are strongly decreased in SLE as a result of classical complement activation. Thirdly, anti-C1q antibodies can be found in approximately 33% of the patients with SLE and usually coincides with classical pathway activation (69, 70).

One of the main functions of C1q is to act as an opsonin to stimulate removal of apoptotic cell fragments. C1q can directly bind to the surface of apoptotic keratinocytes through its globular heads, which results in the formation of C3 convertases. This is followed by cleavage of C3 and the release anaphylatoxin C3a and the opsonin C3b which results in the clearance of apoptotic cells by phagocytosis (71). Furthermore, the collagenous region of C1q can bind to the calreticulin/CD91 complex, which is expressed on the surface of phagocytes. This binding enhances phagocytosis, leading to ingestion of C1q coated apoptotic cells (72). C1q deficiency therefore directly interferes with the clearance of apoptotic cell debris. Although this is interesting from a mechanistic point of view, most SLE patients are C1q sufficient and therefore other mechanisms underlie the impaired clearance of apoptotic debris in SLE. Impaired clearance of apoptotic cells could lead to secondary necrosis, causing disintegration, and high expression of potential auto-antigens on the surface of apoptotic bodies and blebs (73). This induces the production of auto-antibodies against these “lupus auto-antigens” by B-cells. In turn, auto-antibody-antigen complexes bind C1q thereby activating the classical pathway of complement (74). Together with classical pathway activation as a result of C1q bound to apoptotic blebs, this explains the paradox that SLE patient show strong ongoing complement activation with low and sometimes undetectable serum complement levels (secondary hypocomplementemia). Furthermore, it is thought that prolonged exposure of C1q bound to the surface of apoptotic blebs could become antigenic due to impaired clearance that consequently induces production of auto-antibodies to C1q (75). Recent studies have demonstrated that anti-C1q specifically interacts with C1q bound to early apoptotic cells and not to C1q bound to immunoglobulins or immune complexes. Antigen-C1q-anti-C1q complexes induce classical pathway activation also causing secondary hypocomplementemia. Besides, this results in interference of the uptake of apoptotic cells and impaired C1q-dependent phagocytosis (76, 77).

The deposition of auto-antibody-antigen complexes can be visualized in skin biopsies by immunofluorescence in the so-called “lupus band test,” demonstrable as granular or linear deposits of IgG, C3b and occasionally IgA and IgM along the dermo-epidermal junction. The lupus band test can be applied to differentiate SLE from CLE and also as a prognostic parameter for patients with LE. A positive reaction can be observed in both lesional as well as non-lesional skin in SLE. However, in CLE only lesional skin shows a positive lupus band test. More importantly, sun-exposed skin biopsies of healthy individuals may also exhibit a positive lupus band test; therefore, it is essential to perform direct immunofluoresence on sun-protected skin (78). Interestingly, also MBL deposition was recently found in lesional skin of patients with SLE, indicating involvement of the lectin pathway (79).

Lastly, there seems to be no clear difference in the role of complement between SLE and CLE. The complement system, especially C1q, appears to play a crucial role in the pathogenesis of SLE and CLE. The absence of functional C1q can lead to impaired clearance of apoptotic cells, resulting in expression of auto-antigens, and induction of auto-antibody generation. Furthermore, C1q can eventually bind to these surface blebs stimulating the production of auto-antibodies to C1q itself. Despite the increased knowledge concerning the pathogenesis of SLE, it remains a complex disease in which multiple mechanisms are of significance.

## Cutaneous Small Vessel Vasculitis (CSVV)

Cutaneous small vessel vasculitis is defined as inflammation of the postcapillary venules in the skin. CSVV without presence of systemic vasculitis is currently named as single organ CSVV, according to the updated Chapel Hill Nomenclature for Vasculitis (80). There are also other subtypes of CSVV with systemic involvement such as anti-neutrophil cytoplasmic antibodies (ANCA)-associated vasculitis and cryoglobulinemic vasculitis. CSVV is the most common type of vasculitis with an incidence of 15 per million. The majority of the patients are adults with a slight preference for females (81). Over the years, numerous studies have revealed multiple factors taking part in the development of CSVV. The disease seems to be associated with drugs, infections, and systemic disorders such as SLE, RA, or malignancy. However, in many patients there is no identifiable cause and in these cases CSVV is then considered as a primary idiopathic entity (82, 83).

The classical presentation of CSVV usually occurs 7–14 days after exposure to a triggering agent. Palpable purpura are the hallmark of CSVV, and appear as red-purple discolorations on the skin varying from 2 to 5 mm in size. However, CSVV presents as many clinical variants, depending on the severity and duration of the disease including urticaria, pustules, erosions, and ulcers (81).

**Figure 4:**
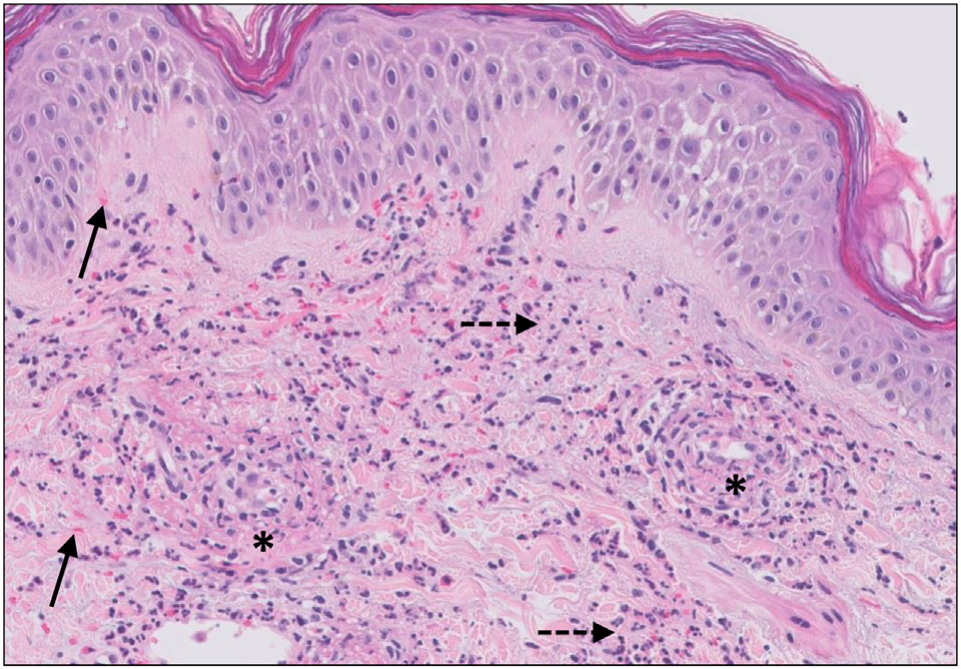
Small vessel leucocytoclastic vasculitis. The section shows all features of leucocytoclastic vasculitis. A mixed inflammatory cell population surrounding the postcapillary venules of the superficial dermis. The infiltrate consists of neutrophils with nuclear dust (dashed arrows) and shows high affinity for the vessels. Features of vascular injury are shown including fibrinoid necrosis (asterisks) and erytrocyt extravasation (solid arrows).

The histopathologic pattern observed in CSVV is leukocytoclastic vasculitis (LCV), which is characterized by a superficial infiltrate surrounding the postcapillary venules predominantly composed of neutrophils, leukocytoclasis, endothelial swelling, extravasation of erythrocytes, and eventually fibrinoid necrosis (Figure 4) (82). Moreover, the presence of eosinophils can be an indicator of drug-induced CSVV.

Accumulating evidence has indicated that the complement system plays a significant role in the pathogenesis of CSVV. Immunofluorescence studies have supported this hypothesis by demonstrating deposits of immunoglobulins of IgM- and IgG classes, C3c and fibrinogen in, and around dermal vessel walls in LCV. Grunwald et al. demonstrate these immunoreactants in skin biopsies of early, mature, and healing stages of vasculitis (84). Detection of vascular IgA deposition is defined for Henoch–Schonlein purpura (85). Vascular immunoglobulin and complement were also detected in non-lesional skin, suggesting that deposition of these immunoreactants is a primary event, and not secondary to endothelial damage (81). In addition, Dauchel and colleagues reported that C3d and C5b-9 were significantly increased in plasma of patients with LCV compared with controls, whereas C3, C1q, C2, C4, and FB were within normal ranges. These findings indicate local as well as systemic complement activation in patients with LCV. Moreover, the increased level of C3d and C5b-9 has been shown to correlate with vasculitis activity. Interestingly, circulating C3d and C5b-9 levels did correlate with disease activity but not with the intensity of perivascular cutaneous depositions, suggesting that C3d and C5b-9 plasma levels are caused by systemic complement activation instead of passive diffusion from cutaneous lesions into the circulation (86).

The most widely accepted concept of the pathogenesis is deposition of immune complexes in postcapillary venules, with inflammation occurring after complement activation. As mentioned previously, multiple etiologies can trigger the formation of circulating immune complexes. These consist of foreign antigens and antibodies in slight excess of antigen, and therefore easily get trapped and subsequently deposited in small vessels. These immune complexes lead to activation of the complement cascade *via* the classical pathway. Among the activated complement products, C5a and C5b-9 seem to be mainly responsible for the endothelial damage observed in CSVV (82, 83). C5a induces activation of neutrophils and upregulation of several adhesion molecules (E-selectin, ICAM-1, and V-CAM) on the surface of endothelial cells, allowing neutrophils, and other inflammatory cells to migrate from the circulation into the site of inflammation (87). Activated neutrophils can secrete reactive oxygen species and also dysregulate the expression of serine proteinases elastase and cathepsin G, which eventually leads to endothelium damage (88). Additionally, decreased tissue plasminogen activator (t-PA) in response to released cytokines due to vascular damage leads to abnormal fibrinolysis and may eventually result in fibrin deposition as reflected on histology (89). Under physiologic conditions, various regulators secreted or/and expressed by endothelial cells maintain control of complement activation. These include C1-INH, FH, FI, vitronectin, clusterin, and CD55/DAF (90). Interestingly, Boom et al. observed that DAF expression on endothelial cells of cutaneous vasculitis was almost completely absent, while DAF expression on intraluminal erythrocytes was unaffected. Expression of DAF on the surface can restrain assembly of the terminal cascade by binding to C3b, and thereby interfering with the C5 convertases. The authors suggest that the absence of DAF might be the consequence of local downregulation of DAF synthesis and might play an important role in the pathogenesis of CSVV (91). However, there is only sparse literature available about potent downregulators of DAF.

In aggregate, the complement system appears to be associated with the development of CSVV. The interaction between complement components and activation products, and the endothelial cells seem to play an essential role in the pro-inflammatory response seen in CSVV. Activation of classical pathway of complement has been recognized in CSVV, regarding to the immune complex-mediated process. In particular, the effects of C5a and MAC on endothelial cells and neutrophils may eventually lead to structural and functional damage of the endothelium resulting in CSVV.

## Urticaria and Urticarial Vasculitis (UV)

### Urticaria

**Figure 5:**
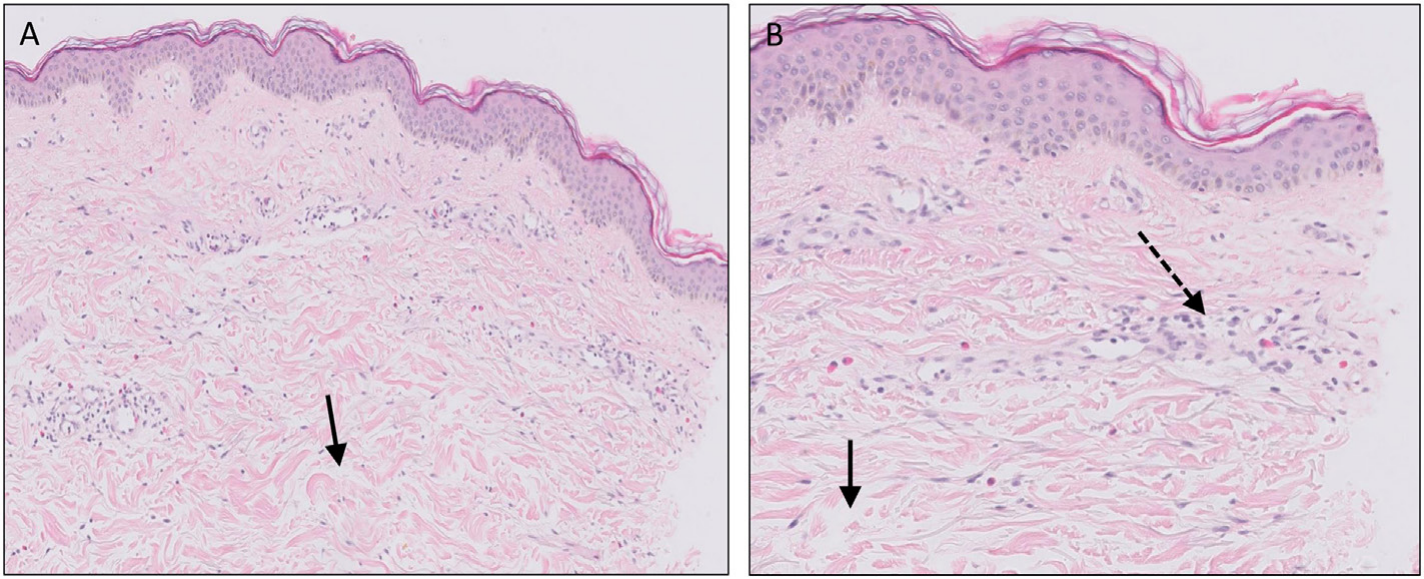
Urticaria. Dermal edema [solid arrows in **(A,B)**] and a sparse superficial predominantly perivascular and interstitial infiltrate of lymphocytes and eosinophils without signs of vasculitis (dashed arrow).

Urticaria is widely held to be one of the most common skin diseases, affecting up to approximately 20% of the population during lifetime. Urticarial lesions are characteristically pruritic, edematous, erythematous papules, or wheals often with pale centers, which can merge into larger plaques and usually resolve within 24 h. Urticaria consists of a wide spectrum ranging from localized wheals to widespread recurrent wheals with angioedema. Coexisting episodes of angioedema appear to be present in approximately 40% of patients with urticaria (92). Angioedema can be characterized as edema in the deep dermal layer and subcutaneous or submucosal tissues. Acute urticaria is defined by disease duration of less than 6 weeks, whereas recurrent urticarial lesions persisting for a period beyond 6 weeks is defined as chronic urticaria (CU). CU can be further divided into chronic inducible urticaria and chronic spontaneous urticaria (CSU). Inducible urticaria is caused by a response to external triggers, including cold, heat, allergens, sunlight, sweat, and pressure (92). CSU is mainly idiopathic, although researchers have identified a subpopulation with an auto-immune etiology. The histopathology of an urticarial lesion mostly involves changes in the upper dermis, consisting of mild dermal edema, and sparse perivascular and interstitial mixed inflammatory infiltrate composed of a variable number of lymphocytes, monocytes, mast cells, eosinophils, and neutrophils (Figure 5) (93). The pathophysiological events in the formation of wheals involve activation of dermal mast cells and basophils by various triggers, including anaphylatoxins C3a and C5a, and also physical stimuli. It is well established that binding of antigen to antigen-specific IgE on mast cells and basophils activates and degranulates these inflammatory cells (type 1 hypersensitivity reaction). The most important active substance in urticaria is histamine. Histamine induces vasodilation, increases vascular permeability, and stimulation of sensory nerve endings leading to pain or itching. Other important mediators such as TNF-α, leukotrienes, platelet-activator, and prostaglandin D2 may also promote an inflammatory response (94). The exact trigger or cause of histamine release has not been identified in most patients with CSU. Evidence of an auto-immune etiology in approximately 45% of patients has been presented. Patients with CSU have shown to possess circulating IgG auto-antibodies directed against the α subunit of IgE (anti-FcεR1α) and IgE receptor in their sera, corresponding to a type 2 hypersensitivity reaction (95). Additionally, increased frequency of thyroid dysfunction and thyroid auto-antibodies (anti-microsomal peroxidase and anti-thyroidglobulin) are found in patients with CSU (96).

IgG auto-antibodies bound to IgE or IgE receptor on mast cells results in the activation of complement *via* the classical pathway followed by generation of C5a and C5b-9. Subsequently, C5a can mediate mast cell activation *via* C5aR1 ligation with subsequent degranulation and release of mediators, resulting in urticarial wheals (97, 98). Accumulated evidence has shown that complement is involved in degranulation of mast cells and basophils in some patients with CSU (97). Firstly, one study demonstrated that heat inactivation of serum complement of patients with CU, decreased the capacity of serum to release histamine from basophils (99). Ferrer and colleagues found that unless serum containing C2 and C5 was added to IgG derived from CU sera, histamine is not released from mast cells, implicating that activation of the classical complement pathway is required for mast cell degranulation (100). In parallel with early studies, the presence of complement, especially C5a, had a role in augmenting IgG-dependent histamine release from basophils (101). Although the immune reaction in urticaria is regarded as a type 1 and type 2 hypersensitivity reaction, also local complement deposition can be found in about one-third of chronic urticarial lesions without signs of systemic activation, suggesting a type 3 hypersensitivity reaction as well (102–105).

### Urticarial Vasculitis

Urticarial vasculitis is a relatively uncommon disease with an estimated prevalence of 5%, occurring more often in women. Clinical cutaneous manifestations of UV consist of erythematous urticarial papules and plaques that last 24–72 h with a tendency to heal with purpura or hyperpigmentation. Patients with UV can be classified as normocomplementemic UV (NUV) or hypocomplementemic UV (HUV) depending on serum complement levels. Although NUV is mostly idiopathic, HUV is associated with a more severe form of disease and can indicate an underlying disease like SLE or hypocomplementemic urticarial vasculitis syndrome (HUVS) (106). HUVS is characterized by urticaria with hypocomplementemia, arthralgia/arthritis, glomerulonephritis, recurrent abdominal pain, and obstructive lung disease (107). According to retrospective observations, there seems to be no transition among UV subtypes (108). Histopathologically, lesions of UV reveal combined features of urticaria with superimposed vascular damage. In contrast to LCV, vascular damage in UV is more subtle with endothelial activation, sparse karyorrhexis, and focal fibrinoid necrosis present in only the minority of cases (109).

Various reports support the hypothesis that UV is an immune complex-mediated disease. Deposits of immunoglobulins, complement, and/or fibrinogen in the vessel walls are often observed by immunofluorescence in patients with UV. Additionally, immune complexes are also regularly detected in the blood circulation of patients with UV. There are numerous etiologies that can trigger the formation of immune complexes in UV, including auto-antibodies, infections, and medications. Formation of immune complexes leads to activation of the complement cascade *via* the classical pathway and subsequently generation of C3a, C5a, and C5b-9. Release of C5a can lead to a chain of events, including activation of neutrophils, mast cell degranulation, and eosinophil degranulation, which in turn can result in endothelial damage (106).

Wisnieski et al. were the first to suggest that the pathogenesis of HUVS might involve abnormal genetic background (110). Subsequently, one study found that genetic mutations in the DNASE1L3 gene are associated with a familial form of HUVS and SLE. The protein encoded by this gene functions as endonuclease, which is possibly responsible for the removal of DNA during apoptosis (111). This finding supports the link between SLE and HUVS. Another similar feature in both diseases is the presence of auto-antibodies against C1q that can be detected in 100% of the patients with HUVS and in approximately one-third of the patients with SLE (69, 108). Anti-C1q antibodies bind to the collagenous region of C1q and usually coincide with decreased levels of classical pathway complement components as a result of complement activation. Additionally, decreased levels of CH50, C2, and C4 were also found in patients with HUVS (106). These findings implicate that complement might contribute to the pathogenesis of HUVS.

To summarize, UV consist of a wide spectrum of cutaneous, systemic features ranging from urticaria with mild vasculitis to systemic vasculitis combined with hypocomplementemia. Classical activation of complement is involved in the pathogenesis of UV and HUVS.

## Bullous Pemphigoid (BP)

In this section, we will review the role of complement in the most common auto-immune bullous dermatosis, BP. Although complement has also been shown to be involved in the pathogenesis of other auto-immune bullous dermatoses, such as mucous membrane pemphigoid, epidermolysis bullosa acquisita, dermatitis herpetiformis, and bullous systemic LE, the reader is referred elsewhere to more detailed publications on this topic (112–114).

**Figure 6:**
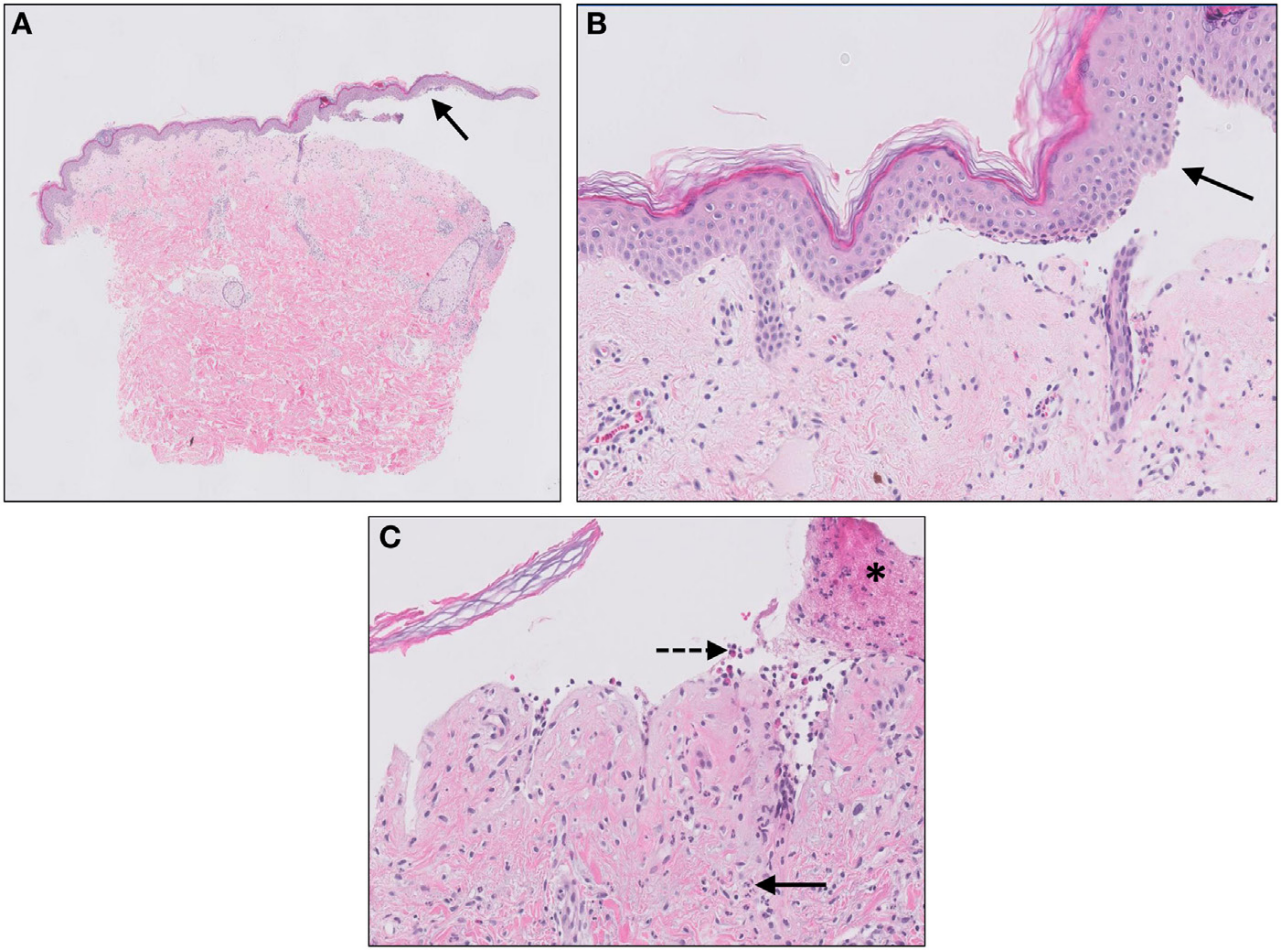
Bullous pemphigoid. Subepidermal blistering [solid arrows in **(A,B)**] and influx of inflammatory cells including eosinophils and neutrophils in the dermis [solid arrow **(C)**] and blister cavity [dashed arrows **(C)**]. In **(C)** also deposition of fibrin is noted (asterisks).

Bullous pemphigoid is the most frequently encountered acquired auto-immune blistering disease most commonly affecting the elderly. Clinically, patients present with tense pruritic dome-shaped fluid filled blisters measuring up to several centimeters in diameter. The most commonly recognized BP antigens are BP180 (BPAG2) and BP230 (BPAG1) and less frequently, antibodies can be found against plectins and LAD1. Most antibodies in BP are directed against the NC16A domain of BP180, which is a major non-collagenous extracellular antigenic site. Separation in BP occurs at the level of the lamina lucida. A skin biopsy from an established blister reveals a subepidermal blister, often accompanied by a superficial dermal infiltrate of lymphocytes, eosinophils, neutrophils, mast cells, and monocytes/macrophages (Figure 6). These inflammatory cells are also found in the blister fluid in the cell-rich variant of BP. Immunofluorescence demonstrates specific linear IgG and/or C3b deposition along the basement membrane zone. Although deposition of complement fragments such as C3c and C1q is routinely used for diagnostic reasons, activation of the complement system has also been shown to play a crucial role in the development of disease.

Complement components and activation fragments including C1q, C3, C3c, C3d, C4, C4d, C5, C5b-9, FB, FH, and properdin have been found at the basement membrane zone and blister fluid in BP. These findings indicate involvement of both classical and alternative pathways in the pathogenesis of BP (115–117). Recently, complement split products C3d and C4d have been shown to be deposited along the basement membrane zone in formalin fixed paraffin embedded tissue and might be a potential substitute for direct immunofluorescence in the near future (118–122).

Liu et al. developed an experimental mouse model of BP using a rabbit antibody against murine BP180NC14A, a homolog of human BP180NC16A, which is, however, very poorly conserved in murine protein. This antibody was passively transferred in neonatal mice and developed a subepidermal blistering disease closely mimicking clinical BP. This experimental model of BP has demonstrated a critical role for complement activation in BP. Mice deficient of C5 or depleted of complement by cobra venom factor showed no clinical or histological evidence of BP in contrast to wild-type (WT) or complement sufficient mice. Also, injection of F(ab’)2 fragments prepared from the pathogenic rabbit anti-murine BP180 did not show induction of BP, indicating a crucial role for the IgG Fc portion in BP. It is the Fc portion of antibody that expresses antigen-binding sites for C1q, thereby initiating classical complement activation (123). Using the same BP model, C4 deficient and WT mice pretreated with anti-mC1q were resistant to develop BP, also strongly indicating a crucial role for classical complement activation in BP. However, FB deficient mice developed delayed and less intense blisters, indicating that also the alternative pathway is involved in BP, probably acting in concert with classical pathway activation *via* the amplification loop (124, 125). The complement-mediated development of BP appeared to be dependent on mast cells and neutrophils. Mice depleted of circulating neutrophils did not develop BP after injection of anti-mBP180. Reconstitution with C5a or injection with neutrophil chemokine IL-8 in C5 deficient mice regained susceptibility to BP (126). These findings indicate that neutrophil recruitment in BP is C5a dependent. However, mice deficient in mast cells or C5aR1 deficient mast cells failed to develop BP, despite the activation of complement and in the presence of neutrophils. The passive transfer model exhibits extensive mast cell degranulation, preceding neutrophil infiltration, and subepidermal blistering. It was found that complement-dependent chemokinesis of neutrophils is, at least in part, dependent on mast cell degranulation *via* C5aR1 (124, 127).

Although interesting, it is questionable whether this mice model can be easily translated to the human situation since BP180NC14a is poorly preserved in mice. Therefore, a humanized mice model was developed in which mouse BP180NC14A was replaced with the homologous human BP180NC16A epitope cluster region. Mice injected with anti-human BP180NC16A subsequently development BP (128). A similar model was developed by Nishie et al. introducing the human COL17 cDNA transgene into Col17-null mice (129). Importantly, the previous findings of complement-dependent development of BP were confirmed (128).

Notably, although complement plays an important role in the pathogenesis of BP, more and more evidence emerges that also complement-independent mechanisms exist. Several groups have shown direct pathogenic effects of auto-antibodies leading to depletion of COL17 in keratinocytes and contributing to skin fragility in a complement-independent manner (130–132).

Most of the previously cited studies were performed in animal or *in vitro* models, making interpretation of the data limited. However, in a large cohort (*n* = 300) patient study, Romeijn et al. have shown that in the majority of BP skin biopsies, complement deposition could be demonstrated. More interestingly, deposition of complement was related to clinical and serological disease activity strengthening the crucial role of complement in this disease (133).

## Complement Inhibiting Strategies in Skin Diseases

For years, many attempts in producing complement-specific drugs have been made with limited success. The main challenge in creating complement-specific drugs has been to discover the balance between sufficient blocking of complement activity in order to prevent local tissue damage and preserving the protection of this system. As outlined in the current review, clinical and experimental studies have demonstrated variable roles of complement in the pathogenesis of skin diseases. The findings and proposed triggers for complement activation in different skin diseases are summarized in Table 1. With the impending recognition of neutrophil driven auto-inflammatory diseases, IL-1β and TNF-α inhibitors have now been proven beneficial for the treatment of specific auto-inflammatory diseases such as the use of adalimumab in the treatment of HS (54). The successful use of these agents is primarily based on the inhibition of neutrophil chemotaxis. As described previously, C5a acts as a potent recruiter of neutrophils by binding to C5aR1 expressed primarily by neutrophils. Moreover, C5a can trigger increased vascular permeability on endothelial cells and activate the adaptive immune system. Therapeutic intervention in C5a–C5aR axis could therefore be a promising target for the treatment of neutrophil driven skin diseases such as psoriasis and HS as well as other neutrophilic dermatoses. Selective neutralization of the C5a–C5aR interaction offers opportunities to inhibit complement activation without interfering with membrane attack complex formation and thereby the defensive potential of complement. A decade ago, C5aR antagonist PMX53 has already been evaluated and trialed in psoriasis with initial encouraging results. Orally administered cyclic peptidomimetic PMX-53 was tested in a phase 1b/2a trial in 10 patients with psoriasis and positive results in many disease measures were obtained. However, unfortunately, this drug has a very short half-life of only 70 min which severely limits its use in clinical practice (134). Besides psoriasis, intervention in C5a–C5aR axis has very recently been shown to beneficial in the treatment of HS. Blockade of C5a by an anti-C5a antibody demonstrated up to 83% HS clinical response rate at the endpoint of anti-C5a treatment in a phase 2 clinical study, indicating the importance of complement in this disease (NCT03001622). Furthermore, a promising new therapeutic agent is a small molecule C5aR inhibitor CCX168 (Avacopan). The drug can be orally administered, shows a relatively long half-life, has shown limited side effects and promising results in other neutrophil driven diseases, for example, in ANCA-associated vasculitis (135). These therapeutic agents are preferred over the use of Eculizumab (Soliris) which is a recombinant humanized monoclonal antibody directed against C5. Eculizumab intercepts the complement cascade at the terminal effector pathway by binding tightly to C5 and preventing cleavage to C5b. Consequently, this suppresses the formation of the C5b-9, increasing susceptibility to infection (136).

**Table 1:** Overview of complement in skin diseases and the proposed triggers of complement activation.

Skin disease	Species	System	Findings related to complement	Proposed triggers of complement activation and/or results	Reference
Psoriasis	Human	Scale extracts	Elevated: C3a, C4a, C5a, C5a-des-Arg	Natural anti-SC antibodies C5 cleavage by serine proteases in SC Microbial products Alternative pathway activation after trauma	Tagami et al. (14, 32) Ohkohchi et al. (17) Schröder et al. (18) Takematsu et al. (19) Mrowietz et al. (16)
	
	Human	Skin biopsies	Deposition: C3b	IgG, IgM, IgG deposition	Weiss et al. (15)
	
	Human	Serum	Elevated: FB, Bb, C3, C3a, C3b, C4, C4a, C4d, C5b-9, C1-INH, C4bBp, FH, FI Decreased: properdin Normal: C3bBb, C1s-C1-INH	Systemic complement is not activated based on normal circulating C3bBb and C1s-C1-INH complexes (20)	Fleming et al. (20) Kapp et al. (21) Ohkohchi et al. (22, 23) Rosenberg et al. (24) Marley et al. (25)
	
	Mice	IMQ psoriasis model, C3−/−	Elevated: C3 gene expression in WT mice after IMQ treatment	Trigger: unknown Results: protection against development of psoriatic phenotype in C3−/− mice	Giacomassi et al. (40)

Acne vulgaris	Human	Skin biopsies	C3b deposition	P. Acnes Natural anti-SC antibodies Immune complexes	Scott et al. (44) Dahl et al. (12)

HS	Human	Skin biopsies	Gene induction of complement pathways	Trigger: unknown	Blok et al. (55)
	
	Human	Serum	Elevated: C5a, C5b-9	PAMPs and DAMPS	Kanni et al. (56)

LE	Human	Genome	Congenital deficiency of C1q, C1r, C1s, C4, C2. C4 copy number variation	Breach of self-tolerance and auto-antibody formation Inefficient clearing of apoptotic cells/debris	Racila et al. (64) Boeckler et al. (65) Chen JY et al. (66)
	
	Human	Serum	Elevated: anti-C1q auto-antibodies	Immune complexes, (anti-C1q) auto-antibodies	Prodeus et al. (67) Antes et al. (69) Hoekzema et al. (70)

CSVV	Human	Skin biopsies	C3c deposition in vessel wall	Immune complexes	Grunwald et al. (84)
	
	Human	Serum	Elevated: C3d, C5b-9	Immune complexes	Dauchel et al. (86)

CU	Human	Serum	Complement-dependent histamine release from basophils and mast cells	Type 2 hypersensitivity reaction: auto-antibodies against IgE-FcεR1α and IgE receptor	Zweiman et al. (99) Ferrer et al. (100)

UV	Human	Serum	Elevated: Anti-C1q auto-antibodies Hypocomplementemia	(Anti-C1q) auto-antibodies, immune complexes	Wisnieski et al. (108)

BP	Human	Skin biopsies Blister fluid	Deposition: Linear basement membrane deposition of C1q, C3b, C3c, C3d, C4, C4d, C5, C5b-9, FB, FH, properdin	Anti-BP180 and BP230 auto-antibodies	Dahl et al. (115) Jordon et al. (116, 117) Chandler et al. (118) Kassaby et al. (119) Magro et al. (120) Pfaltz et al. (121) Villani et al. (122)
	
	Mice	C4−/− C5−/− FB−/− Anti-mC1qCVF depletion	Linear basement membrane C3 deposition in WT animals	Mechanism: anti-BP180 and BP230 auto-antibodies →classical pathway activation, alternative pathway activation (amplification loop)Results in complement −/− mice: protection against development of BP	Liu et al. (123) Heimbach et al. (124) Nelson et al. (125) Liu et al. (128) Nishie et al. (129)

*SC, stratum corneum; IMQ, imiquimod; HS, hidradenitis suppurativa; PAMPs, pathogen-associated molecular patterns; DAMPs, danger-associated molecular patterns; LE, lupus erythematosus; CSVV, cutaneous small vessel vasculitis; CU, chronic urticaria; UV, urticarial vasculitis; BP, bullous pemphigoid*.

Besides therapeutic intervention in C5a–C5aR axis in auto-inflammatory diseases, intervention in complement activation is likely to be beneficial in BP. This review discussed several experimental mouse studies of BP that demonstrated a crucial role of complement activation in this auto-immune bullous disease. Noteworthy, currently two trials with complement inhibitors are ongoing in BP: TNT-009 is a monoclonal antibody against C1s (NCT02502903) while coversin targets C5 and leukotriene B4 (http://adisinsight.springer.com/trials/700284185).

Altogether, intervention in C5a–C5aR axis and the inherent inhibition of neutrophil recruitment might be the most interesting treatment for complement-mediated inflammatory skin diseases.

## Conclusion

The complement system represents far more than the originally thought function as a non-specific defense mechanism against micro-organisms. By virtue of extensive murine model studies and genome-wide association studies, our knowledge regarding the functions and hidden connections of complement has greatly expanded. Besides the protective role of complement, improper regulated activation of complement can lead to extensive tissue damage. Local and systemic complement activation has been demonstrated in several skin diseases; however, whether complement activation has pathogenic significance in different skin diseases remains to be investigated. Although not all triggers of complement activation are fully elucidated in different skin diseases, antibody, and immune complex deposition play an important role in (classical) complement activation. However, earlier and more recent experimental studies using mice deficient in complement components have shown involvement of complement in the pathogenesis of BP and experimental psoriasis. Furthermore, preliminary results of a phase IIa clinical study in HS shows promising results using a monoclonal anti-C5a antibody. These result indicate that complement mediates, at least in part, inflammatory skin diseases, in particular neutrophil driven diseases. Therefore, inhibition of neutrophil recruitment through intervention in the C5a–C5aR axis might be the most interesting target of intervention in inflammatory skin diseases.

## Author Contributions

JG, MS, RR and JD wrote the article. MV, EP, and JD edited and approved the final manuscript.

## Conflict of Interest Statement

The authors declare that the research was conducted in the absence of any commercial or financial relationships that could be construed as a potential conflict of interest.
